# Correction: Bat IFITM3 restriction depends on S-palmitoylation and a polymorphic site within the CD225 domain

**DOI:** 10.26508/lsa.202000747

**Published:** 2020-04-29

**Authors:** Camilla TO Benfield, Farrell MacKenzie, Markus Ritzefeld, Michela Mazzon, Stuart Weston, Edward W Tate, Boon Han Teo, Sarah E Smith, Paul Kellam, Edward C Holmes, Mark Marsh

**Affiliations:** 1 Department of Pathobiology and Population Sciences, Royal Veterinary College, University of London, London, UK; 2 MRC Laboratory for Molecular Cell Biology, University College London, London, UK; 3 Department of Chemistry, Imperial College London, London, UK; 4 Department of Infectious Disease, Imperial College Faculty of Medicine, Wright Fleming Institute, St Mary’s Campus, London, UK; 5 Kymab Ltd, The Bennet Building (B930), Babraham Research Campus, Cambridge, UK; 6 Marie Bashir Institute for Infectious Diseases and Biosecurity, Charles Perkins Centre, School of Life and Environmental Sciences and Sydney Medical School, The University of Sydney, Sydney, New South Wales, Australia

See original article: Bat IFITM3 restriction depends on S-palmitoylation and a polymorphic site within the CD225 domain, 3(1), 2019.

Following publication, the authors realized that the middle name initial of co-author Edward W. Tate was inadvertently missing. This has now been corrected and the authors regret any confusion this may have caused. Both the HTML and PDF versions of the article have been corrected.

## Supplementary Material

Reviewer comments

