# Supplementary material for: Bat IFITM3 restriction depends on S-palmitoylation and a polymorphic site within the CD225 domain
Source: Life Sci Alliance. 2019 Dec 11;3(1):e201900542. doi: 10.26508/lsa.201900542 (PMC6907390; doi:10.26508/lsa.201900542)
Supplement: Supplementary file 1 [file LSA-2019-00542_TableS1.doc]

| **Common Name** | **Species Name** | **Family** | **Sequence identifiers for IFITMs used in selection pressure analysis**  *** identified by cDNA analysis** | **Evidence for function** |
| --- | --- | --- | --- | --- |
| Black flying fox | *Pteropus alecto* | Pteropodidae | IFITM3* | Expressed transcript (PIT), antiviral restriction (unpublished data) |
| Large flying fox | *Pteropus vampyrus* | Pteropodidae | XM_011386598 (IFITM3) | Expressed, intron present |
| Greater mouse-eared bat | *Myotis myotis* | Vespertilionidae | IFITM3*  IFITM1* | Antiviral restriction [39]  Expressed transcript containing intron (RACE) |
| Little brown bat | *Myotis lucifugus* | Vespertilionidae | XM_006108167 (IFITM3)  XM_006108170 (IFITM1) | Expressed, intron present  Expressed, intron present |
| David’s Myotis | *Myotis davidii* | Vespertilionidae | XM_006761354 (IFITM1) | Expressed, intron present |
| Serotine bat | *Eptesicus serotinus* | Vespertilionidae | IFITM3* | Expressed transcript containing intron (RACE) |
| Greater spear-nosed bat | *Phyllostomus hastatus* | Phyllostomidae | IFITM3* | Expressed transcript containing intron (RACE) |
| Silky short-tailed bat | *Carollia brevicauda* | Phyllostomidae | IFITM3* | Expressed transcript containing intron (RACE) |
| Pig | *Sus scrofa* | Suidae | NM_001201382.1 (IFITM3)*  XM_003354415 (IFITM1-like)* | Antiviral restriction [39]  Expressed transcript containing intron (RACE) |
| Cow | *Bos taurus* | Bovidae | NM_181867 (IFITM3)  XM_024987403 (IFITM1) | Expressed [91] |
| Sheep | *Ovis aries* | Bovidae | XM_004019741 (IFITM3) | Antiviral restriction [42] |
| Mouse | *Mus musculus* | Muridae | IFITM1, IFITM2, IFITM3,  XM_011246029 (IFITM7) | Antiviral restriction [21] |
| Human | *Homo sapiens* | Hominidae | IFITM1, IFITM2, IFITM3 | Antiviral restriction [21] |
| Gorilla | *Gorilla gorilla gorilla* | Hominidae | XM_004050339 (IFITM1)  XM_004050342 (IFITM2)  XM_004050337 (IFITM3) | Antiviral restriction (unpublished data) |
| Chimpanzee | *Pan troglodytes* | Hominidae | XM_009459613 (IFITM1)  NM_001198767 (IFITM2)  NM_001198757 (IFITM3) | Antiviral restriction (unpublished data) |
| Orangutan | *Pongo abelii* | Hominidae | NM_001198762 (IFITM1)  XM_002821311 (IFITM3) | Antiviral restriction (unpublished data) |
| Colobus monkey | *Colobus angolensis palliatus* | Cercopithecidae | XM_011961854 (IFITM3) | Antiviral restriction [46] |

Supplementary Table1. IFITM genes used for selection pressure analysis.

Mammalian IFITM genes used in the analysis of selection pressures, their sequence identifiers, accession numbers (if available) and evidence for function are shown. Chiropteran species are marked in red. An asterisk (*) denotes genes identified from our cDNA analysis using rapid amplification of cDNA ends (RACE) or proteomics informed by transcriptomics (PIT).
