# Supplementary material for: Correction: Bat IFITM3 restriction depends on S-palmitoylation and a polymorphic site within the CD225 domain
Source: Life Sci Alliance. 2020 Apr 29;3(6):e202000747. doi: 10.26508/lsa.202000747 (PMC7195047; doi:10.26508/lsa.202000747)
Supplement: Reviewer comments [file LSA-2020-00747_review_history.pdf]

Life Science Alliance

Correction: Bat IFITM3 restriction depends on S-palmitoylation and a polymorphic site within the...

Camilla Benfield, Farrell Mackenzie, Markus Ritzefeld, Michela Mazzon, Stuart Weston, Edward Tate, Boon Han Teo, Sarah Smith, Paul Kellam, Edward Holmes, and Mark Marsh
DOI: https://doi.org/10.26508/lsa.202000747

Corresponding author(s): Camilla Benfield, Royal Veterinary College

**Review Timeline:**	Submission Date:	2020-04-20
	Accepted:	2020-04-20

Scientific Editor: Andrea Leibfried

Transaction Report:

(Note: With the exception of the correction of typographical or spelling errors that could be a source of ambiguity, letters and reports are not edited. The original formatting of letters and referee reports may not be reflected in this compilation.)

April 20, 2020

RE: Life Science Alliance Manuscript #LSA-2020-00747

Dr. Camilla Tamsin Olivia Benfield
Royal Veterinary College
Hawkshead Lane
Hatfield, Hertfordshire AL9 7TA
United Kingdom

Dear Dr. Benfield,

Thank you for submitting your Correction entitled "Correction: Bat IFITM3 restriction depends on S-palmitoylation and a polymorphic site within the...".

The final published version of your corrected manuscript will be deposited by us to PubMed Central upon online publication.

Sincerely,

Andrea Leibfried, PhD
Executive Editor
Life Science Alliance
Meyerhofstr. 1
69117 Heidelberg, Germany
t +49 6221 8891 502
e a.leibfried@life-science-alliance.org
www.life-science-alliance.org

April 20, 2020

RE: Life Science Alliance Manuscript #LSA-2020-00747

Dr. Camilla Tamsin Olivia Benfield
Royal Veterinary College
Hawkshead Lane
Hatfield, Hertfordshire AL9 7TA
United Kingdom

Dear Dr. Benfield,

Thank you for submitting your Correction entitled "Correction: Bat IFITM3 restriction depends on S-palmitoylation and a polymorphic site within the...".

The final published version of your corrected manuscript will be deposited by us to PubMed Central upon online publication.

Sincerely,

Andrea Leibfried, PhD
Executive Editor
Life Science Alliance
Meyerhofstr. 1
69117 Heidelberg, Germany
t +49 6221 8891 502
e a.leibfried@life-science-alliance.org
www.life-science-alliance.org
